# Deformable image registration and interobserver variation in contour propagation for radiation therapy planning

**DOI:** 10.1120/jacmp.v17i3.6110

**Published:** 2016-05-08

**Authors:** Adam C. Riegel, Jeffrey G. Antone, Honglai Zhang, Prachi Jain, Jagdeep Raince, Anthony Rea, Angelo M. Bergamo, Ajay Kapur, Louis Potters

**Affiliations:** ^1^ Department of Radiation Medicine Northwell Health Lake Success NY USA; ^2^ Hofstra Northwell School of Medicine Hempstead NY USA

**Keywords:** interobserver variation, deformable image registration, B‐spline, contour propagation

## Abstract

Deformable image registration (DIR) and interobserver variation inevitably introduce uncertainty into the treatment planning process. The purpose of the current work was to measure deformable image registration (DIR) errors and interobserver variability for regions of interest (ROIs) in the head and neck and pelvic regions. Measured uncertainties were combined to examine planning margin adequacy for contours propagated for adaptive therapy and to assess the trade‐off of DIR and interobserver uncertainty in atlas‐based automatic segmentation. Two experienced dosimetrists retrospectively contoured brainstem, spinal cord, anterior oral cavity, larynx, right and left parotids, optic nerves, and eyes on the planning CT (${\text{CT}}_{1}$) and attenuation‐correction CT of diagnostic PET/CT (${\text{CT}}_{2}$) for 30 patients who received radiation therapy for head and neck cancer. Two senior radiation oncology residents retrospectively contoured prostate, bladder, and rectum on the postseed‐implant CT (${\text{CT}}_{1}$) and planning CT (${\text{CT}}_{2}$) for 20 patients who received radiation therapy for prostate cancer. Interobserver variation was measured by calculating mean Hausdorff distances between the two observers' contours. ${\text{CT}}_{2}$ was deformably registered to ${\text{CT}}_{1}$ via commercially available multipass B‐spline DIR. ${\text{CT}}_{2}$ contours were propagated and compared with ${\text{CT}}_{1}$ contours via mean Hausdorff distances. These values were summed in quadrature with interobserver variation for margin analysis and compared with interobserver variation for statistical significance using two‐tailed *t*‐tests for independent samples (α=0.05). Combined uncertainty ranged from 1.5‐5.8 mm for head and neck structures and 3.1‐3.7 mm for pelvic structures. Conventional 5 mm margins may not be adequate to cover this additional uncertainty. DIR uncertainty was significantly less than interobserver variation for four head and neck and one pelvic ROI. DIR uncertainty was not significantly different than interobserver variation for four head and neck and one pelvic ROI. DIR uncertainty was significantly greater than interobserver variation for two head and neck and one pelvic ROI. The introduction of DIR errors may offset any reduction in interobserver variation by using atlas‐based automatic segmentation.

PACS number(s): 87.57.nj, 87.55.D‐

## I. INTRODUCTION

Deformable image registration (DIR) is increasingly being incorporated into radiation therapy. Applications include multimodality image registration,^(1)^ atlas‐based automatic segmentation,^(2)^ dose summation,^(3)^ and contour propagation for online,^4^, ^5^ and offline^4^, ^6^ adaptive radiation therapy. Unlike conventional “rigid” registration, DIR does not assume spatial invariance between all voxels of both image sets. By using complex mathematical models, such as optical flow^7^, ^8^ or B‐splines,^2^, ^9^ DIR stretches one image set to match another at a local (often voxel‐by‐voxel) level. This is useful in anatomical regions that have many degrees of freedom, such as the neck, or that are prone to change over time. Such changes could occur in daily cycles (e.g., the bladder or rectum) or progressively over the course of treatment (e.g., soft tissue in the head and neck that change with weight loss).

In radiation therapy planning, DIR is often applied to two CT image sets. The first CT is usually the CT “simulation” acquired in the treatment position. The second CT could be a diagnostic or prior simulation CT. Deformable image registration between CTs can sometimes be useful in itself (diagnostic CT with intravenous contrast, for example), but when the second CT is DICOM‐linked to secondary data, such as the PET portion of a PET/CT or a prior treatment plan, data can be transformed with the deformation vector field just like the CT to which they are linked. This process enables contour propagation from one CT to another. Clinically, physician‐drawn contours are often propagated from simulation CT to cone‐beam CT or resimulation CT to adapt treatment^(6)^ or from a multipatient CT “atlas” for automatic segmentation.^(2)^ For atlas‐based automatic segmentation, CTs and their associated clinical contours are added to the CT atlas. When a new patient is to be segmented automatically, the algorithm searches the atlas for a CT which best matches the clinical CT, DIR is performed, and atlas contours are propagated to the new CT.

Studies have assessed the accuracy of DIR algorithms using digital phantoms,^(10)^ physically deforming phantoms,^(11)^ mathematical descriptors,^12^, ^13^ and clinical CT scans.^14^, ^15^, ^16^ Digital^(10)^ or physically deforming phantom studies,^(11)^ while useful, may lack clinical complexity. Mathematical descriptors, such as curl and the Jacobian, have been proposed as useful metrics to quantify the deformation vector field.^12^, ^13^ Though such descriptors could be beneficial in the future, they are untested clinically and lack intuitive clinical meaning. Landmark‐based quality assurance from clinical CT currently represents the most robust quantification of DIR accuracy. Castillo et al.^(14)^ demonstrate the efficacy of landmark pairs to assess DIR quality in thoracic CT imaging and suggest the technique could be used for routine DIR quality assurance. In a large multi‐institutional study, Brock et al.^(16)^ measure DIR error for intra‐ and intermodality DIR using landmarks and found DIR errors on the order of voxel size.

Though much attention has been justifiably focused on DIR accuracy, the influence of interobserver variation on DIR uncertainty in regard to landmark identification is nonnegligible.^(17)^ The same may be true for contour propagation in adaptive therapy or atlas‐based automatic segmentation. For adaptive therapy, where contour propagation is used to reduce the contouring burden on the physician and dosimetrist, propagated volumes would presumably include uncertainties associated with both DIR and interobserver contouring variability. Currently, neither is commonly included in planning target volume (PTV) or planning organ‐at‐risk volume (PRV) margins. For atlas‐based automatic segmentation where the use of DIR has been reported to reduce interoberver contouring variation,^(18)^ the trade‐off between this benefit and the uncertainty introduced by DIR error has not been quantified.

The purpose of the current work was to evaluate the influence of interobserver variation in contours propagated by deformable image registration. Two analyses were performed. First, the uncertainties associated with interobserver variation and DIR were measured for normal tissue contours in a sample of patients in two anatomical sites and a potential margin expansion was evaluated; second, the magnitude of DIR uncertainty was compared with interobserver variation.

## II. MATERIALS AND METHODS

Thirty (30) head and neck and 20 prostate cancer patients who received radiation therapy at our institution were retrospectively included in the study. Site‐specific methodology is described in the next sections.

### A. Head and neck

Head‐and‐neck patients were retrospectively included if CT simulation was accompanied by diagnostic PET/CT used for treatment planning. Per our standard clinical CT simulation protocol, patients were immobilized using five‐point thermoplastic masks (Orfit Industries, Wijnegem, Belgium) and scanned with the Philips Brilliance Big Bore CT scanner (Philips Medical Systems, Milpitas, CA). Simulation CT scans employed helical acquisition, 120 kVp, 3 mm slice thickness, and 65–70 cm reconstructed field of view. Images were reconstructed using filtered back‐projection. PET/CT scanning was performed within the institution for 18 of 30 patients with 120 kVp, 3.75 mm slice thickness, and 50 cm reconstructed field of view. The remaining scans were acquired from outside institutions with tube voltages between 120–140 kVp, slice thicknesses ranging from 3–5 mm, and reconstructed fields of view ranging from 244 cm to 700 cm. All CT scans utilized automatically modulated tube current and 512 by 512 image matrices. No immobilization was used for any diagnostic PET/CT scan and a curved tabletop was utilized for all patients.

Two dosimetrists with substantial head and neck planning experience were asked to independently contour brainstem, spinal cord, anterior oral cavity, larynx, right and left parotids, right and left optic nerves, and right and left eyes on the simulation CT scan (${\text{CT}}_{1}$). Anterior oral cavity was defined as the region splitting the base of tongue between the hard palate and glossopharyngeal sulcus. Larynx was defined as the superior edge of the epiglottis to inferior edge of the cricoid cartilage. Spinal cord was defined from the inferior edge of the brainstem to the superior edge of sternum.

After one month (to reduce memory bias), both dosimetrists contoured the same regions of interest (ROI) on the CT portion of the diagnostic PET/CT scan (${\text{CT}}_{2}$). One iteration of mutual information‐based rigid registration and multipass B‐spline DIR was used to register ${\text{CT}}_{2}$ to ${\text{CT}}_{1}$. Contouring and DIR was performed in Velocity software version 3.0.0 (Varian Medical Systems, Atlanta, GA). The CT portion of the PET/CT scan was used in this study because PET/CT‐to‐CT‐simulation represents the majority of DIR in our department and presents a challenging anatomical match due to lack of immobilization on the PET/CT.

### B. Prostate

Prostate patients were retrospectively included if CT simulation for external beam and postimplant CT for prostate seed implant was performed. Simulation CT was acquired via helical acquisition with 140 kVp, 3 mm slice thickness, and 60 cm field of view. Postimplant CT was performed three to four weeks after implantation and was acquired via helical acquisition, 120 kVp, 3 mm slice thickness, and 20 cm field of view. All CT scans utilized automatically modulated tube current, filtered back‐projection reconstruction, 512 by 512 image matrices, and flat table tops.

Two senior radiation oncology residents were asked to independently contour prostate, rectum, and bladder on the postimplant CT scan (${\text{CT}}_{1}$). Rectum was defined as 1 cm above and below the prostate.

One month later (to reduce memory bias), the residents contoured the same ROIs on the external beam simulation CT (${\text{CT}}_{2}$). The same deformable technique described above was used to register ${\text{CT}}_{2}$ to ${\text{CT}}_{1}$. If excessive bladder/rectum filling caused visibly misregistered contours after one iteration of DIR, an additional iteration was performed by reducing the DIR region to focus on the bladder and/or rectum. Postimplant CT was used in this study because registration of postimplant CT to external beam CT simulation may facilitate composite external beam and brachytherapy dose summation.^(19)^

### C. Analysis

Regions of interest were generically termed ${\text{RO}}_{\text{ij}}$ where the first subscript signifies the dosimetrist (1 or 2) and the second subscript signifies the image set (${\text{CT}}_{1}$ or ${\text{CT}}_{2}$). ${\text{ROI}}_{12}$ and ${\text{ROI}}_{22}$ were transformed via the DIR vector field to ${\text{CT}}_{1}$, resulting in ${\text{ROI}}_{1D}$ and ${\text{ROI}}_{2D}$. The variation between the observers' contours on ${\text{CT}}_{1}$ (${\text{ROI}}_{11}$ vs. ${\text{ROI}}_{21}$) was the interobserver variation ($V_{\text{IO}}$). The variation between the original ${\text{CT}}_{1}$ contours and the deformed ${\text{CT}}_{2}$ contours (${\text{ROI}}_{11}$ vs. ${\text{ROI}}_{1D}$ and ${\text{ROI}}_{21}$ vs. ${\text{ROI}}_{2D}$) was termed the total measured variation ($V_{T}$).

**Figure 1:**
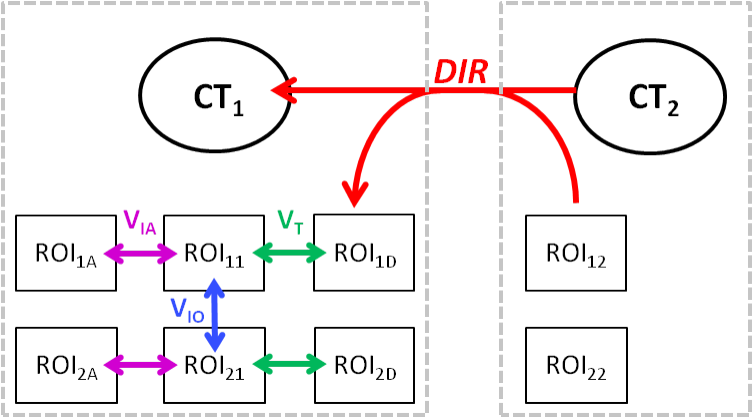
Schematic representation of the relationships between regions of interest (ROIs). ROIs on the left and right are contoured on ${\text{CT}}_{1}$ and ${\text{CT}}_{2}$, respectively. ${\text{ROI}}_{\text{ij}}$ represents structures contoured by observer i on image set j. ${\text{ROI}}_{12}$ and ${\text{ROI}}_{22}$ are deformed via the DIR vector field to form ${\text{ROI}}_{1D}$ and ${\text{ROI}}_{2D}$. $V_{T}$ is the total variation between the original ${\text{CT}}_{1}$ contours (${\text{ROI}}_{11}$ and ${\text{ROI}}_{21}$) and deformed ${\text{CT}}_{2}$ contours (${\text{ROI}}_{1D}$ and ${\text{ROI}}_{2D}$). $V_{\text{IO}}$ is the interobserver variation measured between contours drawn on ${\text{CT}}_{1}$ by both observers. $V_{\text{IA}}$ is the intraobserver variation measured between contours drawn on ${\text{CT}}_{1}$ for each observer.

If DIR worked perfectly and the observers were able to replicate the ROIs exactly on ${\text{CT}}_{1}$ and ${\text{CT}}_{2}$, the variation between ${\text{ROI}}_{i1}$ and ${\text{ROI}}_{\text{iD}}$ should be zero. In practice, $V_{T}$ contained two components: Error associated with the DIR technique ($E_{\text{def}}$) and intraobserver variation ($V_{\text{IA}}$) because each ROI was drawn once on ${\text{CT}}_{1}$ and again on ${\text{CT}}_{2}$. $V_{\text{IA}}$ could not be explicitly measured for all patients due to time limitations of the participants. Instead, all observers recontoured the same ROIs on ${\text{CT}}_{1}$ for five patients approximately one month after ${\text{CT}}_{2}$ contour completion. These ROIs were termed ${\text{ROI}}_{1A}$ and ${\text{ROI}}_{2A}$ and were compared to ${\text{ROI}}_{11}$ and ${\text{ROI}}_{21}$, respectively, to determine m${\text{ROI}}_{\text{IA}}$. Table 1 summarizes the quantities and their definitions, and Fig. 1 schematically represents the relationships between them.

**Table 1:** Contour names, measured quantities, and definitions.

*Quantity*	*Definition*
${\text{ROI}}_{\text{ij}}$	Structures contoured by observer *i* on image set *j*
${\text{ROI}}_{\text{iD}}$	Structures contoured by observer *i* deformed from ${\text{CT}}_{2}$ to ${\text{CT}}_{1}$
${\text{ROI}}_{\text{iA}}$	Structures recontoured by observer *i* on ${\text{CT}}_{1}$
${\text{ROI}}_{\text{IO}}$	Interobserver variation: deviation between observer 1 and observer 2 contours on ${\text{CT}}_{1}$
${\text{ROI}}_{\text{IA}}$	Intraobserver variation: deviation between observer *i* original contours and observer *i* recontours on ${\text{CT}}_{1}$
${\text{ROI}}_{T}$	Total variation: deviation between observer *i* contours and deformed observer *i* contours on ${\text{CT}}_{1}$; contains both intraobserver variation and residual deformable registration errors
$E_{\text{def}}$	Residual deformable registration error: intraobserver variation subtracted in quadrature from total variation
$E_{\text{def}2}$	Residual deformable registration error after second iteration of deformable registration

An adaptation of the three‐dimensional Hausdorff distance, the mean variation between two surfaces, described by Varadhan et al.,^(20)^ was used to quantify $V_{\text{IO}},V_{\text{IA}}$, and $V_{T}$ for each ROI. The Hausdorff calculation was performed for each point in the primary ROI against all points in the secondary ROI to determine the closest distance between the two surfaces in three dimensions. The mean distance over all points was calculated to represent the average variation between the two surfaces. The calculation was performed with a built‐in function in the Velocity software.

Interobserver variation ($V_{\text{IO}}$) was calculated and averaged over all patients for each ROI. Total variation ($V_{T}$) was calculated and averaged over all patients for each ROI for each observer. The sample size of $V_{T}$ was thus twice the sample size of $V_{\text{IO}}$ as two observers are required to calculate ${\text{ROI}}_{\text{IO}}$. Intraobserver variation ($V_{\text{IA}}$) was averaged over the five randomly chosen patients described above for each ROI in each anatomical site. Because we measured $V_{\text{IA}}$ for a sample of patients and not each patient individually, a linear sum of $V_{\text{IA}}$ and $E_{\text{def}}$ uncertainties could not be assumed. Instead, we assumed $V_{\text{IA}}$ would be less than $V_{\text{IO}}$,^21^, ^22^ and $V_{\text{IA}}$ and $E_{\text{def}}$ behaved like population‐based margins and were summed in quadrature,^(23)^ and $E_{\text{def}}$ could be calculated using the following equation:

(1) $$E_{def}=\sqrt{V_{T}{\;}^{2}-V_{IA}{\;}^{2}}$$

To estimate the margin expansion required to account for both interobserver variation and DIR, we summed the average $E_{\text{def}}$ and $V_{\text{IO}}$ in quadrature for each region of interest. We used Student's *t*‐tests for independent samples (α=0.05) to compare means of $E_{\text{def}}$ and $V_{\text{IO}}$ distributions for statistical significance for each ROI.

## III. RESULTS

### A. Head and neck

All 10 ROIs were contoured for 27 of 30 patients. The larynx and left parotid were not contoured due to surgical removal before radiation therapy for one patient each. The left parotid was not contoured due to proximity to the primary tumor for one patient. Table 2 displays means and standard deviations of $V_{T}$ ${\text{ROI}}_{\text{IA}}$, $E_{\text{def}}$, and $V_{\text{IO}}$ for 10 ROIs. Intraobserver variation was less than interobserver variation for all structures. Table 3 shows the quadrature sum of $V_{\text{IO}}$ and $E_{\text{def}}$ for potential margin expansion for each ROI. There was notable variation in the combined uncertainty for the head and neck ROIs, ranging from 1.5 mm for the eyes to 5.8 mm for the anterior oral cavity.

**Figure 2:**
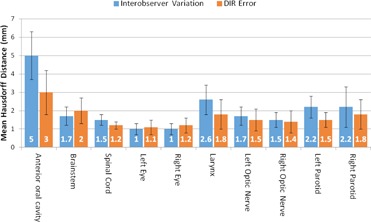
Comparison of interobserver variation and deformable image registration (DIR) error for head and neck regions of interest. Values represent the mean Hausdorff distance calculated between the two surfaces for all patients. Error bars represent 1 SD.

**Figure 3:**
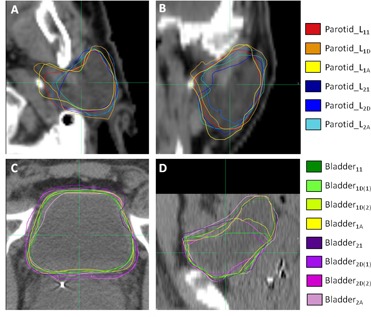
Axial (a) and coronal (b) slices of left parotid contours for one patient. Axial (c) and sagittal (d) slices of bladder contours for one patient. Subscripts follow the definition in the text. Note the bladder has two deformed contours to represent the first and second pass of the deformable image registration algorithm.

Figure 2 compares interobserver variation ($V_{\text{IO}}$) and residual DIR errors ($E_{\text{def}}$). Error bars represent 1 SD. $E_{\text{def}}$ was significantly less than $V_{\text{IO}}$ for the anterior oral cavity, spinal cord, larynx, and left parotid. $E_{\text{def}}$ was not significantly different than $V_{\text{IO}}$ for the brainstem, right parotid, left and right optic nerves. $E_{\text{def}}$ was significantly greater than $V_{\text{IO}}$ for the left and right eyes, but the difference was less than 0.5 mm. Left parotid contours for one patient are shown in Fig. 3(a) (axial) and 3(b) (coronal) for comparison.

**Table 2:** Total variation ($V_{T}$), intraobserver variation ($V_{\text{IA}}$), residual deformation errors ($E_{\text{def}}$), and interobserver variation ($V_{\text{IO}}$) for head and neck anatomy.

	*Total Variation*		*Intraobserver Variation*		*Residual Deformable Registration Errors*		*Interobserver Variation*	
*Structure*	*N*	$V_{T}\;(\text{mm})$	$V_{\text{IA}}\;(\text{mm})$	$E_{\text{def}}\;(\text{mm})$	$V_{\text{IO}}\;(\text{mm})$
Anterior Oral Cavity	60	3.7±1.3	10	2.3±0.6	60	3.0±1.2	30	5.0±1.3
Brainstem	60	2.4±0.8	10	1.3±0.4	60	2.0±0.7	30	1.7±0.5
Cord	60	1.5±0.3	10	0.9±0.2	60	1.2±0.2	30	1.5±0.3
Left Eye	60	1.4±0.5	10	0.7±0.3	60	1.1±0.4	30	1.0±0.3
Right Eye	60	1.4±0.5	10	0.7±0.3	60	1.2±0.4	30	1.0±0.3
Larynx	58	2.3±1.1	10	1.5±0.8	58	1.8±0.8	29	2.6±0.8
Left Optic Nerve	60	1.6±0.7	10	0.4±0.2	60	1.5±0.6	30	1.7±0.5
Right Optic Nerve	60	1.6±0.8	10	0.7±0.5	60	1.4±0.6	30	1.5±0.4
Left Parotid	56	2.2±0.5	10	1.6±0.3	56	1.5±0.4	28	2.2±0.6
Right Parotid	60	2.3±0.8	10	1.4±0.3	60	1.8±0.8	30	2.2±1.1

N = represents the number of contours analyzed.

**Table 3:** Quadrature sum of residual deformation errors ($E_{\text{def}}$) and interobserver variation ($V_{\text{IO}}$) for all regions of interest.

*Structure*	*Potential Expansion (mm)*
Anterior Oral Cavity	5.8
Brainstem	2.6
Cord	1.9
Left Eye	1.5
Right Eye	1.5
Larynx	3.2
Left Optic Nerve	2.6
Right Optic Nerve	2.8
Left Parotid	2.6
Right Parotid	2.8
Prostate	3.7
Bladder	3.1
Rectum	3.6

### B. Prostate

All three ROIs were contoured for all 20 patients. Table 4 shows means and standard deviations of $V_{T},V_{\text{IA}}$, and $E_{\text{def}}$ for three ROIs. Bladder and rectum have an additional comparison ($E_{\text{def}2}$) for the additional pass of the DIR algorithm (seven and four patients, respectively). Table 3 shows the quadrature sum of $V_{\text{IO}}$ and $E_{\text{def}}$ or $E_{\text{def}2}$ (if applicable) for potential margin expansion. The combined uncertainty for prostate yielded a narrower range than head and neck; bladder demonstrated combined uncertainty of 3.1 mm and rectum yielded 3.7 mm.

**Figure 4:**
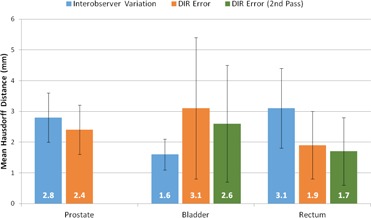
Comparison of interobserver variation and deformable image registration (DIR) error for male pelvic regions of interest. Values represent the mean Hausdorff distance calculated between the two surfaces for all patients. Error bars represent 1 SD.

Figure 4 compares interobserver variation ($V_{\text{IO}}$) and residual DIR errors ($E_{\text{def}}$ and $E_{\text{def}2}$). Error bars represent 1 SD. $E_{\text{def}}$ was significantly less than $V_{\text{IO}}$ for rectum, was not significantly different than $V_{\text{IO}}\text{for}$ prostate, and was significantly more than $V_{\text{IO}}\text{for}$ bladder by 1.5 mm. A second iteration of DIR focused on the bladder or rectum decreased deformation errors ($E_{\text{def}2}$) by 16.8% for bladder and 10.8% for rectum. $E_{\text{def}2}$, however, remained significantly greater than $V_{\text{IO}}\text{for}$ bladder and significantly less than $V_{\text{IO}}$ for rectum. Bladder contours for one patient are shown in Figs. 3(c) (axial) and 3(d) (coronal) for illustrative comparison.

**Table 4:** Total variation ($V_{T}$), intraobserver variation ($V_{\text{IA}}$), residual deformation errors ($E_{\text{def}}$ & $E_{\text{def}(2)}$), and interobserver variation ($V_{\text{IO}}$) for male pelvic anatomy.

	*Total Variation*		*Intraobserver Variation*		*Residual Deformable Registration Errors*		*Residual Deformable Registration Errors (* $2^{nd}$ *pass)*		*Interobserver Variation*	
*Structure*	*N*	$V_{T}\;(\text{mm})$	*N*	$V_{\text{IA}}\;(\text{mm})$	*N*	$E_{\text{def}}\;(\text{mm})$	*N*	$E_{\text{def}(2)}$	*N*	$V_{\text{IO}}\;(\text{mm})$
Prostate	40	3.0±1.0	10	1.8±0.6	40	2.4±0.8	0	20	2.8±0.8
Bladder	40	3.4±2.3	10	1.3±0.4	40	3.1±2.3	14@@2.6±1.9	20	1.6±0.5
Rectum	40	3.2±1.6	10	2.5±1.1	40	1.9±1.1	8@@1.7±1.1	20	3.1±1.3

N = represents the number of contours analyzed.

## IV. DISCUSSION

The current work examines DIR errors ($E_{\text{def}}$) in contour propagation and interobserver variation ($V_{\text{IO}}$) in contour delineation for a sample of patients in two anatomical sites. The analysis was applied in two ways: first, to suggest a margin expansion for combined uncertainty of interobserver variation and DIR, and second, to directly compare interobserver variation and DIR uncertainty.

Numerous publications have suggested appropriate PTV and PRV margins for three‐dimensional conformal and intensity‐modulated radiation therapy, ranging from 2‐5 mm for head and neck, depending on immobilization and frequency of image guidance^24^, ^25^, ^26^, ^27^, ^28^, ^29^, ^30^, ^31^, ^32^ and 3‐10 mm for prostate depending on frequency of image guidance.^33^, ^34^, ^35^ Some authors have suggested the conventional 5 mm margin for head and neck is conservative and margin reduction may be possible,^24^, ^25^, ^27^, ^29^ but others have reported local setup uncertainties meet or exceed 5 mm.^26^, ^28^, ^36^

Our study indicates combined uncertainty of interobserver variation and DIR ranged from 1‐6 mm for head and neck structures and between 3‐4 mm for pelvic structures. Assuming that appropriate PRV margins can be conservatively extrapolated from PTV margin data, the combination of 2‐3 mm reported setup uncertainty for head and neck, interobserver variation, and DIR error in quadrature would yield margins less than 5 mm for all but the anterior oral cavity, suggesting that conventional margins may be sufficient to cover the additional uncertainty of interobserver variation. This assumes, of course, the lower estimates for setup uncertainty, which may not be valid for anatomical subregions within the head and neck.^26^, ^28^, ^36^ For prostate, the quadrature sum including interobserver variation and DIR would only be covered by a 5 mm margin for the lower reported setup uncertainty of 3 mm. Rasch et al.,^(37)^ however, suggest that margins including delineation variability (with no consideration for DIR) should be between 7.9‐9.7 mm for head and neck and 6.1‐9.5 mm for prostate — substantially larger than our hypothetical margin which would include interobserver variation and DIR. The authors note, however, that this margin is an overestimation given the lack of increase in recurrences with increasingly conformal therapy.^(37)^ Gordon and Siebers^(38)^ suggest that calculated prostate PTV margins are conservative because dosimetric margins extend beyond the nominal PTV expansion.

In the head and neck region, DIR uncertainty was significantly less than interobserver variation for 4 of 10 ROIs, not significantly different for 4 of 10 ROIs, and significantly greater than interobserver variation for 2 of 10 ROIs. In the pelvic region, DIR uncertainty was significantly less than interobserver variation for 1 of 3 ROIs, not significantly different for 1 of 3 ROIs, and significantly greater than interobserver variation for 1 of 3 ROIs. The current work is similar to a recent study by Hoffmann et al.^(39)^ comparing DIR accuracy to interobserver variation for a sample of head and neck and abdominal patients using 30‐50 landmark points delineated by five observers on planning and treatment CT images. The authors found interobserver variation in landmark definition to be 1.2±1.1 mm and residual misalignment after B‐spine DIR to be between 1‐4 mm for 50% of landmarks. Although we compared surface separation between ROIs rather than points, our head and neck interobserver and DIR variability measurements compare favorably with the published results. Our analysis suggests that atlas‐based segmentation using DIR may introduce normal tissue contour errors on par with interobserver variation for some anatomical structures, diminishing the advantage of observer‐independent, atlas‐based automatic segmentation. The increase in workflow efficiency, however, may be worthwhile given the net uncertainty remains relatively constant.

There are a few limitations to the current work. First, the current study only considers contour propagation. Surface analyses^(20)^ or overlap metrics^(40)^ provide limited information about deformation accuracy within a structure, so results of this study should not be generalized to other DIR applications, such as dose summation.^(41)^ Second, the study was limited to a single commercially available DIR algorithm, though the methodology employed should be transferrable to other DIR algorithms. Propagated contours are saved as DICOM data, and can be exported and analyzed with surface separation metrics in third‐party commercial software or software developed in‐house.^(42)^ Future work will include more observers for more robust interobserver analysis, and will focus on abnormal anatomy such as tumor and target volumes which are important for dose summation and adaptive radiation therapy. Mencarelli et al.^(43)^ found that B‐spline DIR performs worse with tumor borders. Mohamed et al.^(15)^ compared deformed target volumes to manually segmented reference target volumes and found 95% Hausdorff distances between 5‐10 mm.

## V. CONCLUSIONS

Deformable image registration and interobserver variation influence contour propagation using a commercially available B‐spine deformable image registration algorithm. Deformable image registration uncertainty was significantly less than, or not significantly different from, interobserver variation for most ROIs in the male pelvic and head and neck regions. Use of deformable image registration for atlas‐based automatic segmentation may introduce uncertainty on par with interobserver variation. Combined interobserver variation and deformable image registration uncertainty may exceed conventional planning margins.

## ACKNOWLEDGMENTS

The author (AR) would like to thank Eric Klein and David Collingridge for their help with this paper.

## COPYRIGHT

This work is licensed under a Creative Commons Attribution 4.0 International License.
